# Translation into portuguese of a set of questionnaires designed to evaluate the impact of using a telepresence robot during postoperative ward rounds

**DOI:** 10.1590/0100-6991e-20223377-en

**Published:** 2022-09-28

**Authors:** LEONARDO KRISTEM, CACIO RICARDO WIETZYCOSKI, GUILHERME DA SILVA MAZZINI

**Affiliations:** 1 - Hospital de Clínicas de Porto Alegre, Serviço de Cirurgia do Aparelho Digestivo - Porto Alegre - RS - Brasil; 2 - Hospital da Unimed do Vale do CaÍ, CEMTrOM - Centro Especializado por Excelência para o Tratamento da Obesidade e Metabolismo - Montenegro - RS - Brasil

**Keywords:** Telemedicine, Questionnaires, Translating, Post-Operative Care, Telemedicina, Questionários, Tradução, Cuidado Pós-Operatório

## Abstract

**Introduction:**

the use of telepresence grows with the advancement of technology integration into medical practice. Regarding surgery, effective distance communication can translate into better perioperative care. Though, the patients’ perception about this modality needs to be critically evaluated. Structured questionnaires using objective scales are the instruments of choice for measuring subjective aspects. However, there are no such questionnaires available in Portuguese. Our objective was, thus, translate and adapt into Portuguese a specific questionnaire evaluating the use of telepresence robots during post-operatory ward rounds.

**Methods:**

search on PubMed and selection of a set of validated questionnaires in English, translation into Portuguese according to the principles of good practice for the translation and cultural adaptation process for patient-reported outcomes measures. The original author approved the final version.

**Results:**

three questionnaires that assess ward rounds assisted by a telepresence robot were translated. Questionnaires are directed to the patient who receives the visit via telepresence or face-to-face visit and to the medical team accompanying the visit. The questionnaires use the Likert scale and contain thirteen questions that address 5 spheres: Communication; Dignity and Confidentiality; Contents; Time; General Impressions.

**Conclusions:**

this is the first instrument in Portuguese designed to assess the impression of patients and professionals involved in ward rounds using a telepresence robot. It has the potential to be used in clinical studies involving the use of this technology in care.

## RESUMO

**Introdução:**

O uso da telepresença cresce com o avanço da integração de tecnologias à prática médica. No que tange a cirurgia, a comunicação à distância efetiva pode traduzir-se em um melhor cuidado perioperatório. No entanto, a percepção das partes acerca desta modalidade carece de avaliação científica. Questionários estruturados através de escalas são os instrumentos de escolha na mensuração de aspectos subjetivos. Entretanto, não há uma ferramenta validada neste molde em língua portuguesa. Nosso objetivo foi traduzir e adaptar para língua portuguesa um questionário específico de percepção sobre o uso de robôs de telepresença em visita hospitalar pós-cirúrgica.

**Métodos:**

foi realizada busca no PubMed e selecionado um conjunto de questionários validados em língua inglesa. Após, realizada a tradução para a língua portuguesa conforme as boas práticas para o processo de tradução e adaptação cultural. A autora original avaliou e aprovou a versão traduzida.

**Resultados:**

foram traduzidos três questionários autoaplicáveis e direcionados para os pacientes que recebem a visita por telepresença ou presencial e à equipe médica que acompanha as visitas com o objetivo de avaliar a visita hospitalar assistida por robô de telepresença. O conjunto utiliza a escala de Likert e contêm treze perguntas que abordam as esferas Comunicação; Dignidade e Confidencialidade; Conteúdo; Tempo; Impressões Gerais.

**Conclusões:**

este é o primeiro instrumento em língua portuguesa desenhado para avaliar a impressão de pacientes e profissionais envolvidos em rounds utilizando um robô de telepresença, tendo potencial para ser utilizado em estudos clínicos que envolvam a utilização desta tecnologia na assistência.

## INTRODUCTION

Telemedicine is the use of technology employed via communication networks for the delivery of health services, assistance, and medical education from one geographic location to another^1^. Its use and its modalities, such as telepresence, have increased on a large scale with the advancement of the incorporation of technologies into medical practice, associated with the need to optimize costs and resources. Since 2020, the use of telemedicine has grown exponentially due to the SARS-CoV-2 pandemic and the need to integrate policies such as social distancing and better use of human resources^2^. Similarly, several previous experiences have demonstrated positive outcomes for medical staff, institutions, and patients using telemedicine^3^ ^,^ ^4^. Even in eminently traditional activities such as surgery, studies have been conducted in recent decades investigating the role of telemedicine and its potential to generate benefits without interfering with patient safety. In a study conducted with 23 high-risk patients undergoing bariatric surgery who received preoperative evaluation and follow-up via teleconference, the authors found equivalent surgical outcomes when compared with matched patients who had traditional care^5^. Despite its dissemination, however, the subjective perception of the parties about this type of interaction still lacks scientific evaluation^6^.

Questionnaires structured through scales or scores are the instruments of choice for measuring subjective aspects^7^, allowing their use in scientific studies in a reproducible and comparable manner. So far, there are no structured questionnaires available in Portuguese aiming to assess the impact of using telepresence technology in hospitalized patients that could be applied in clinical studies.

The objective of the present study was to select and translate into Portuguese a group of questionnaires, already available and validated in English, which perform an objective and reproducible assessment of the impact of telepresence robots in postoperative hospital visits, so that these tools can be applied in studies with Portuguese-speaking patients.

## METHODS

### Questionnaire selection

We carried out a search on PubMed and selected an original work by Croghan, Carroll, Reade, Gillis, and Ridgway, 2018^8^. In it, the authors developed and validated a group of questionnaires that assess the perception of patients and medical staff in relation to postoperative medical visits during hospitalization. In this work, patients in postoperative recovery were divided into two groups: one group receiving medical visits from the traditional care team (surgeon accompanied by non-medical staff physically present) and one group receiving visits where the surgeon used a telepresence robot, accompanied by physically present non-medical staff. To assess the subjective perceptions of patients in both groups and the care team, three multidimensional questionnaires were developed, addressing the spheres of Communication, Dignity and Confidentiality, Content, Time, and General Impressions.

### Portuguese language translation

1. Based on a previously used and known translation methodology protocol into Portuguese^9^ ^,^ ^10^, and in accordance with good practices for the process of translation and cultural adaptation^11^, the process of translation into Portuguese of the questionnaires used in the original work involved the following steps:
2. Selection: Initially, we performed a search on PubMed and selected the pioneering set of questionnaires in English, designed, validated, and published by Croghan, Carroll, Reade, Gillis, and Ridgway, 2018^8^;
3. Preparation: we requested authorization by the author of the original tool, granting her a license to use the translated questionnaires;
4. Translation: translation of the questionnaires into Portuguese by two Brazilian researchers fluent in English, adapting the language level to high school education. Each translated the questionnaire independently, seeking a conceptual translation rather than a literal one;
5. Consensus: After the translation phase, the work team, composed of three Brazilian researchers, met and selected the most appropriate version of the set of questionnaires. This was called “Version 1”;
6. Back-translation: A native English speaker, fluent in Portuguese, and experienced in translating medical papers, performed the translation of Portuguese ‘Version 1’ into English, the original language of the set of questionnaires. This professional did not have access to the original English version of the questionnaires and was instructed to perform a literal translation. This was called the “Back-translated Version”;
7. Review and approval by the original author: The Back-Translated Version was submitted for evaluation by the author of the original questionnaires in English, who considered it equivalent to the original version, approving it without objections or requests for modifications;
8. Final report: We wrote a report on the process of translating and adapting the set of questionnaires. This article originates from the report.

Face validity, aiming at an assessment of the perception of the patient undergoing a hospital visit by telepresence, was weighted at each stage in a consensual way among the researchers. As it is not a questionnaire of patient-reported outcomes and because there was rigor in all stages of translation, the researchers judged that there was no need for a validation stage of cognitive unfolding with patients, in accordance with good practices described by Wild, Grove, Martin, Eremenco, McElroy, Verjee-Lorenz, et al., 2005^10^.

## RESULTS

At the end of the review phase, the authors defined the final version of three translated and previously validated questionnaires to be used as clinical research tools evaluating the postoperative hospital visit assisted by a telepresence robot: Questionnaire A (Table 1), aimed at control patients who receive a face-to-face visit; Questionnaire B (Table 2) for the patients who receives the telepresence visit; and Questionnaire C (Table 3) for the medical team accompanying the visit. The questionnaires use a 5-point Likert scale (strongly agree, agree, neutral, disagree, strongly disagree) and contain 13 questions that address the following spheres: Communication, Dignity and Confidentiality, Content, Time, and General Impressions. Questionnaire B also has two additional questions in the General Impressions domain, which assesses patients’ satisfaction and comfort regarding the hospital visit via telepresence.

**Table 1:** Questionnaire A.

Questionário ao paciente				
Nós estamos ansiosos para receber sua opinião sobre a visita à enfermaria. Ficaríamos gratos se você pudesse completar com honestidade o questionário abaixo relacionado aos diferentes aspectos da sua experiência. Por favor, para cada questão circule a resposta que mais reflita sua opinião. Todas as respostas são anônimas.				
Comunicação				
1. Você pôde ver seu médico?				
Concordo plenamente	Concordo	Neutro	Discordo	Discordo Totalmente
2. Você pôde se comunicar com seu médico?				
Concordo plenamente	Concordo	Neutro	Discordo	Discordo Totalmente
3. Você foi cumprimentado(a) pelo seu médico e perguntado(a) sobre o seu bem-estar?				
Concordo plenamente	Concordo	Neutro	Discordo	Discordo Totalmente
4. Você entendeu o que o médico disse para você?				
Concordo plenamente	Concordo	Neutro	Discordo	Discordo Totalmente
5. Você sentiu que podia discutir quaisquer sintomas ou problemas que o incomodavam?				
Concordo plenamente	Concordo	Neutro	Discordo	Discordo Totalmente
Dignidade e confidencialidade				
6. Você sentiu que o(s) médico(s) mantiveram a sua confidencialidade durante a visita à enfermaria?				
Concordo plenamente	Concordo	Neutro	Discordo	Discordo Totalmente
7. A sua dignidade foi preservada durante a visita à enfermaria?				
Concordo plenamente	Concordo	Neutro	Discordo	Discordo Totalmente
8. A equipe comunicou efetivamente seus sinais vitais e resultados de exames ao seu médico assistente/sênior?				
Concordo plenamente	Concordo	Neutro	Discordo	Discordo Totalmente
Conteúdo				
9. A equipe examinou você de maneira apropriada e comunicou os achados ao meu médico assistente/sênior?				
Concordo plenamente	Concordo	Neutro	Discordo	Discordo Totalmente
10. O médico informou você sobre as suas condições?				
Concordo plenamente	Concordo	Neutro	Discordo	Discordo Totalmente
11. Você ficou satisfeito(a) com o plano de manejo?				
Concordo plenamente	Concordo	Neutro	Discordo	Discordo Totalmente
12. Os médicos responderam às minhas questões?				
Concordo plenamente	Concordo	Neutro	Discordo	Discordo Totalmente
Tempo				
13. Eu senti que os médicos ficaram uma quantidade de tempo apropriada comigo?				
Concordo plenamente	Concordo	Neutro	Discordo	Discordo Totalmente

**Table 2:** Questionnaire B.

Questionário ao paciente				
Você foi visitado na enfermaria pelo seu médico assistente/sênior que não estava presente fisicamente, mas que se comunicou com você e com a equipe através de um computador (referido abaixo como “visita à enfermaria por telecomunicação”). Nós estamos ansiosos para receber sua opinião sobre este conceito. Ficaríamos gratos se você pudesse completar com honestidade o questionário abaixo relacionado aos diferentes aspectos da sua experiência. Por favor, para cada questão circule a resposta que mais reflita sua opinião. Todas as respostas são anônimas.				
Comunicação				
1. Você pôde ver seu médico?				
Concordo plenamente	Concordo	Neutro	Discordo	Discordo Totalmente
2. Você pôde se comunicar com seu médico?				
Concordo plenamente	Concordo	Neutro	Discordo	Discordo Totalmente
3. Você foi cumprimentado(a) pelo seu médico e perguntado(a) sobre o seu bem-estar?				
Concordo plenamente	Concordo	Neutro	Discordo	Discordo Totalmente
4. Você entendeu o que o médico disse para você?				
Concordo plenamente	Concordo	Neutro	Discordo	Discordo Totalmente
5. Você sentiu que podia discutir quaisquer sintomas ou problemas que o incomodavam?				
Concordo plenamente	Concordo	Neutro	Discordo	Discordo Totalmente
Dignidade e confidencialidade				
6. Você sentiu que o(s) médico(s) mantiveram a sua confidencialidade durante a visita à enfermaria?				
Concordo plenamente	Concordo	Neutro	Discordo	Discordo Totalmente
7. A sua dignidade foi preservada durante a visita à enfermaria?				
Concordo plenamente	Concordo	Neutro	Discordo	Discordo Totalmente
8. A equipe comunicou efetivamente seus sinais vitais e resultados de exames ao seu médico assistente/sênior?				
Concordo plenamente	Concordo	Neutro	Discordo	Discordo Totalmente
Conteúdo				
9. A equipe examinou você de maneira apropriada e comunicou os achados ao meu médico assistente/sênior?				
Concordo plenamente	Concordo	Neutro	Discordo	Discordo Totalmente
10. O médico informou você sobre as suas condições?				
Concordo plenamente	Concordo	Neutro	Discordo	Discordo Totalmente
11. Você ficou satisfeito(a) com o plano de manejo?				
Concordo plenamente	Concordo	Neutro	Discordo	Discordo Totalmente
12. Os médicos responderam às minhas questões?				
Concordo plenamente	Concordo	Neutro	Discordo	Discordo Totalmente
Tempo				
13. Eu senti que os médicos ficaram uma quantidade de tempo apropriada comigo?				
Concordo plenamente	Concordo	Neutro	Discordo	Discordo Totalmente
Impressões Gerais				
14. Você acha que a visita à enfermaria por telecomunicação é uma solução satisfatória caso seu médico assistente não possa estar presente fisicamente no hospital?				
Concordo plenamente	Concordo	Neutro	Discordo	Discordo Totalmente
15. Se você for internado em um hospital no futuro, você se sentiria confortável com visitas regulares à enfermaria por telecomunicação?				
Concordo plenamente	Concordo	Neutro	Discordo	Discordo Totalmente
16. Por favor liste quaisquer comentários que você tenha, incluindo preocupações ou possíveis áreas para melhoria:				

**Table 3:** Questionnaire C.

Questionário para o médico/enfermeiro da equipe				
Nós estamos muito interessados em receber sua opinião sobre a visita à enfermaria assistida por robô. Tendo participado de duas ou mais dessas visitas, você poderia por favor completar o questionário abaixo a respeito das suas impressões da interação com o médico comunicando-se via robô?				
Comunicação				
1. Você e o(a) paciente puderam ver o médico?				
Concordo plenamente	Concordo	Neutro	Discordo	Discordo Totalmente
2. Você e o(a) paciente puderam se comunicar com o médico?				
Concordo plenamente	Concordo	Neutro	Discordo	Discordo Totalmente
3. Você sentiu que o(a) paciente pôde discutir quaisquer sintomas ou problemas que o incomodavam?				
Concordo plenamente	Concordo	Neutro	Discordo	Discordo Totalmente
4. A equipe comunicou efetivamente os sinais vitais/exame ao seu médico assistente/sênior?				
Concordo plenamente	Concordo	Neutro	Discordo	Discordo Totalmente
5. Você pôde fazer as perguntas que tinha em relação ao cuidado com paciente, via robô?				
Concordo plenamente	Concordo	Neutro	Discordo	Discordo Totalmente
Dignidade e confidencialidade				
6. Você sentiu que a confidencialidade do paciente foi mantida na visita à enfermaria?				
Concordo plenamente	Concordo	Neutro	Discordo	Discordo Totalmente
7. A dignidade do paciente foi preservada durante a visita à enfermaria?				
Concordo plenamente	Concordo	Neutro	Discordo	Discordo Totalmente
Conteúdo				
8. Você e o(a) paciente foram apropriadamente informados do plano de manejo?				
Concordo plenamente	Concordo	Neutro	Discordo	Discordo Totalmente
9. Você ficou satisfeito(a) com o diagnóstico e plano de manejo?				
Concordo plenamente	Concordo	Neutro	Discordo	Discordo Totalmente
Tempo				
10. Você sentiu que foi dispendida uma quantidade de tempo apropriada com o(a) paciente?				
Concordo plenamente	Concordo	Neutro	Discordo	Discordo Totalmente
Impressões gerais				
11. Você acha que a visita à enfermaria por telecomunicação é uma solução satisfatória caso seu médico assistente não possa estar presente fisicamente no hospital?				
Concordo plenamente	Concordo	Neutro	Discordo	Discordo Totalmente
12. Você se sentiria confortável com visitas regulares à enfermaria por telecomunicação?				
Concordo plenamente	Concordo	Neutro	Discordo	Discordo Totalmente
Impressões Gerais				
13. Por favor liste quaisquer preocupações que você possa ter em relação às visitas à enfermaria por telecomunicação:				

All questionnaires have an explanatory header, customized for each target group, in simple and objective language, allowing the self-application of the instrument. Questionnaires B and C have an open question with a free answer to allow spontaneous comments about the telepresence modality.

The term “ward round” was translated and adapted to “visita à enfermaria” (“ward visit”), for better understanding by the patients. The term “round”, despite being part of the medical jargon in Brazilian Portuguese, could not be understood by most of the target population of the questionnaire and, because of that, it was not chosen for the translation. This choice was corroborated in the back-translation when “visita à enfermaria” was translated back as “ward round”. In the translation phase, in a consensual way among the researchers, we decided to use “plenamente” (“completely”, “fully”) as a translation for “strongly”, despite the literal translation “fortemente”, due to the consecrated use in Portuguese of this adverb associated with the verb “concordar” (“agree”), being more accurate to represent the intent of completeness. Still during this phase, the researchers chose to use gender morphemes with their proper inflections in parentheses in terms with gender marks, according to the standard norm of the Portuguese language.

The original author of the questionnaires fully approved the final translation without objections. 

## DISCUSSION

The present study sought to translate and adapt a set of questionnaires about the subjective perception of patients in hospital visits assisted by telepresence, adopting a protocol previously used in this type of translation^12^. Instead of creating a tool for this purpose, we decided to adapt an already used and validated one, for efficiency and because there is already an existing one in English, allowing its comparative use internationally. Questionnaires like these are essential for investigations using telepresence technology. The use of structured tools such as this one allows a better understanding of subjective aspects, such as the patient’s experience in a hospital or the perception of their care, transferring different domains of health to an objective form, through a numerical scale, the Likert one. In addition, it also allows comparison between individuals and groups, with others or with each other in a given period^7^. For this, it is necessary to ensure adequate understanding of the parts, simplicity, and speed in execution. The structuring of the three questionnaires in this work according to the Likert scale and their synthetic format, in common language, guarantee these characteristics. In addition, the reference selected for this work, originally in English and applied to a British cohort of individuals in postoperative hospital admission, is unprecedented and recent, addressing universal health domains, facilitating its application in different regions. The translation and adaptation of this tool into Portuguese, according to the aforementioned aspects for all questions, was prepared in multiple meetings by the authors, with back-translation by a professional native speaker of English and fluent in Portuguese, with experience in translating documents and scientific medical works, in addition to the contribution and endorsement of the original author.

During the execution of this work, we followed the principles and parameters established in the guidelines for the translation and adaptation of questionnaires. Following these procedures, we guarantee contextual, conceptual, and semantic equivalence, generating a set of questionnaires corresponding to the original. This was the first translation and adaptation of the tool by Croghan, Carroll, Reade, Gillis, and Ridgway, 2018^8^, into a language other than English. 

The translation of questionnaires has intrinsic limitations and known biases, such as cultural differences, the use of technical jargon, and the formatting of the questionnaire itself^12^. We tried to minimize these biases by avoiding the use of medical or ambiguous terms or double questions, always adapting to the educational level of the target population. We kept the original formatting of the questionnaire with the spacing and horizontal alignment of the answers to avoid horizontal bias, as well as the original scale, to avoid inconsistency. Moreover, the number of pages, words, and characters of each questionnaire remained similar. Because it is not a questionnaire dedicated to clinical outcomes reported by patients, considering the face validity, comprehensibility, and clarity analyzed at each stage of the translation, and because the target audience is extremely broad and diverse (patients in a hospital), we deemed necessary to carry out a cognitive unfolding for the elaboration of the final version of this tool.

Notably, the daily practice of medicine values the reduction of costs and time, without reducing the quality of care. In this sense, telemedicine presents itself as an exciting alternative, but like any new medical technology and, in accordance with current health regulations, caution and extensive investigation of its impact are required prior to its adoption. Thus, the present tool adds to the current literature as it has broad applicability potential in the development of telemedicine in Brazil.

## CONCLUSION

This is the first instrument in Portuguese designed to measure the subjective assessment of patients and professionals involved in postoperative hospital visits using a telepresence robot. Critical evaluation of the use of this new technology is essential for it to be incorporated, considering not only its impact on outcomes, but also the perception of patients and hospital staff of the quality of care provided.

We hope that this tool will be widely used in clinical studies involving the use of this technology in healthcare.
